# Application of apoptosis-related genes in a multiomics-related prognostic model study of gastric cancer

**DOI:** 10.3389/fgene.2022.901200

**Published:** 2022-08-05

**Authors:** Chengfei Xu, Zilin Liu, Chuanjing Yan, Jiangwei Xiao

**Affiliations:** ^1^ Chengdu Medical College, Chengdu, China; ^2^ School of Clinical Medicine, Chengdu Medical College, Chengdu, China

**Keywords:** gastric cancer, risk score, prognosis, prediction, immunotherapy sensitivity

## Abstract

Gastric cancer (GC) is one of the most common tumors in the world, and apoptosis is closely associated with GC. A number of therapeutic methods have been implemented to increase the survival in GC patients, but the outcomes remain unsatisfactory. Apoptosis is a highly conserved form of cell death, but aberrant regulation of the process also leads to a variety of major human diseases. As variations of apoptotic genes may increase susceptibility to gastric cancer. Thus, it is critical to identify novel and potent tools to predict the overall survival (OS) and treatment efficacy of GC. The expression profiles and clinical characteristics of TCGA-STAD and GSE15459 cohorts were downloaded from TCGA and GEO. Apoptotic genes were extracted from the GeneCards database. Apoptosis risk scores were constructed by combining Cox regression and LASSO regression. The GSE15459 and TCGA internal validation sets were used for external validation. Moreover, we explored the relationship between the apoptosis risk score and clinical characteristics, drug sensitivity, tumor microenvironment (TME) and tumor mutational burden (TMB). Finally, we used GSVA to further explore the signaling pathways associated with apoptosis risk. By performing TCGA-STAD differential analysis, we obtained 839 differentially expressed genes, which were then analyzed by Cox regressions and LASSO regression to establish 23 genes associated with apoptosis risk scores. We used the test validation cohort from TCGA-STAD and the GSE15459 dataset for external validation. The AUC values of the ROC curve for 2-, 3-, and 5-years survival were 0.7, 0.71, and 0.71 in the internal validation cohort from TCGA-STAD and 0.77, 0.74, and 0.75 in the GSE15459 dataset, respectively. We constructed a nomogram by combining the apoptosis risk signature and some clinical characteristics from TCGA-STAD. Analysis of apoptosis risk scores and clinical characteristics demonstrated notable differences in apoptosis risk scores between survival status, sex, grade, stage, and T stage. Finally, the apoptosis risk score was correlated with TME characteristics, drug sensitivity, TMB, and TIDE scores.

## Introduction

Gastric cancer (GC) is the fifth most commonly diagnosed malignancy in the world with over one million new cases estimated to occur each year (Bray et al., 2018; Smyth et al., 2020). The high mortality rate of GC is attributed to the absence of symptoms in the early stages, and most patients are already at an advanced stage when diagnosed (Digklia and Wagner 2016). There is much research on the pathogenesis of GC, but the pathogenesis is remains unclear. A number of factors have a significant role in increasing the risk of developing GC, such as Helicobacter pylori infection, family history, diet, and alcohol consumption (Machlowska et al., 2020). Currently, GC is treated with a combination of mainly surgical procedures. However, despite the application of neoadjuvant or adjuvant chemotherapy, immune-targeted therapy and surgical treatment, the overall survival of GC patients is still dismal (Tan 2019). Indeed, GC is the third leading cause of cancer-related deaths (Bray et al., 2018). AJCC TNM staging is the most commonly used staging method in GC (Neves Filho et al., 2017; Ji et al., 2018). Patients with the same stage of GC and similar treatment often have different prognoses (Shao et al., 2016), which may be related to the high heterogeneity of the disease. Therefore, it is essential to explore novel tools to predict prognosis and treatment efficacy.

Over the past few decades, researchers have identified many forms of regulated cell death (RCD), such as necrosis, apoptosis, ferroptosis, pyroptosis, and autophagy (Xu et al., 2021; Tsvetkov et al., 2022). For example, miR-522, secreted by cancer-associated fibroblasts (CAFs), has been found to inhibit ferroptosis by targeting ALOX15 in GC (Zhang et al., 2020a). In addition, valproic acid, an anticonvulsant drug, has been reported to inhibit HDAC1/2 and triggered autophagy in GC (Sun et al., 2020). Apoptosis is broadly referred to as programmed cell death, which does not cause an inflammatory response, and Kerr et al. (1972) first used the term “apoptosis” in 1972 to describe a unique form of cell death in 1972. Apoptosis plays a vital role in normal cell turnover, immune cell homeostasis, treatment resistance, and embryonic development (Druwe et al., 2015; Voss and Strasser 2020; Zheng et al., 2020). Aberrant apoptosis can lead to diverse pathologies, such as excessive or limited number of apoptotic cells or the occurrence of apoptosis at an incorrect time or location (Voss and Strasser 2020). Based on many studies, promoting apoptosis has become a promising treatment option that can lead to cancer cell death. N-myc downstream-regulated gene 2 (NDRG2) is a positive regulator of apoptosis that increases tumor sensitivity to anticancer drugs, delays tumor progression and inhibits metastasis (Kim et al., 2021). In addition, many genes have been identified as regulators of apoptosis, including THBS1 (Zhu et al., 2019), TP53 (Napolitano et al., 2020) and MUC16 (Matte et al., 2014), which are closely associated with tumorigenesis and progression. Caspase-3 has been shown to activate nuclear translocation of YAP and regulate cell proliferation and organ size (Yosefzon et al., 2018). Moreover, prostaglandin E2 (PGE2), which is secreted by apoptotic cells mediates the stimulation of cancer proliferation (Fogarty and Bergmann 2017). *In vitro* assays have shown that elevated FAT4 expression leads to decreased proliferation, migration, invasiveness and neovascularization of CRC cells (Pan et al., 2020), and Junfen et al. found that sortilin-related VPS10 domain-containing receptor one and cubilin inhibit the development of cervical adenocarcinoma (Xu and Lu 2021). Nevertheless, the molecular basis of apoptosis and its function in cancer prognosis and therapy are still poorly understood and need to be further explored.

In the present study, we generated an apoptosis risk score model for 23 genes associated with apoptosis that can distinguish the association between gene expression and GC prognosis, and we validated the model using TCGA and GEO datasets. This model may provide a new reference for prognosis prediction in GC. We also confirmed the correlation of the apoptosis risk score with clinical case characteristics, immune cell infiltration, tumor mutation burden and drug sensitivity. Overall, we generated a predictive model for GC prognosis and also verified its potential function in the development and progression of the disease.

## Materials and methods

### Sources of data and clinical characteristics

**FIGURE 1:**
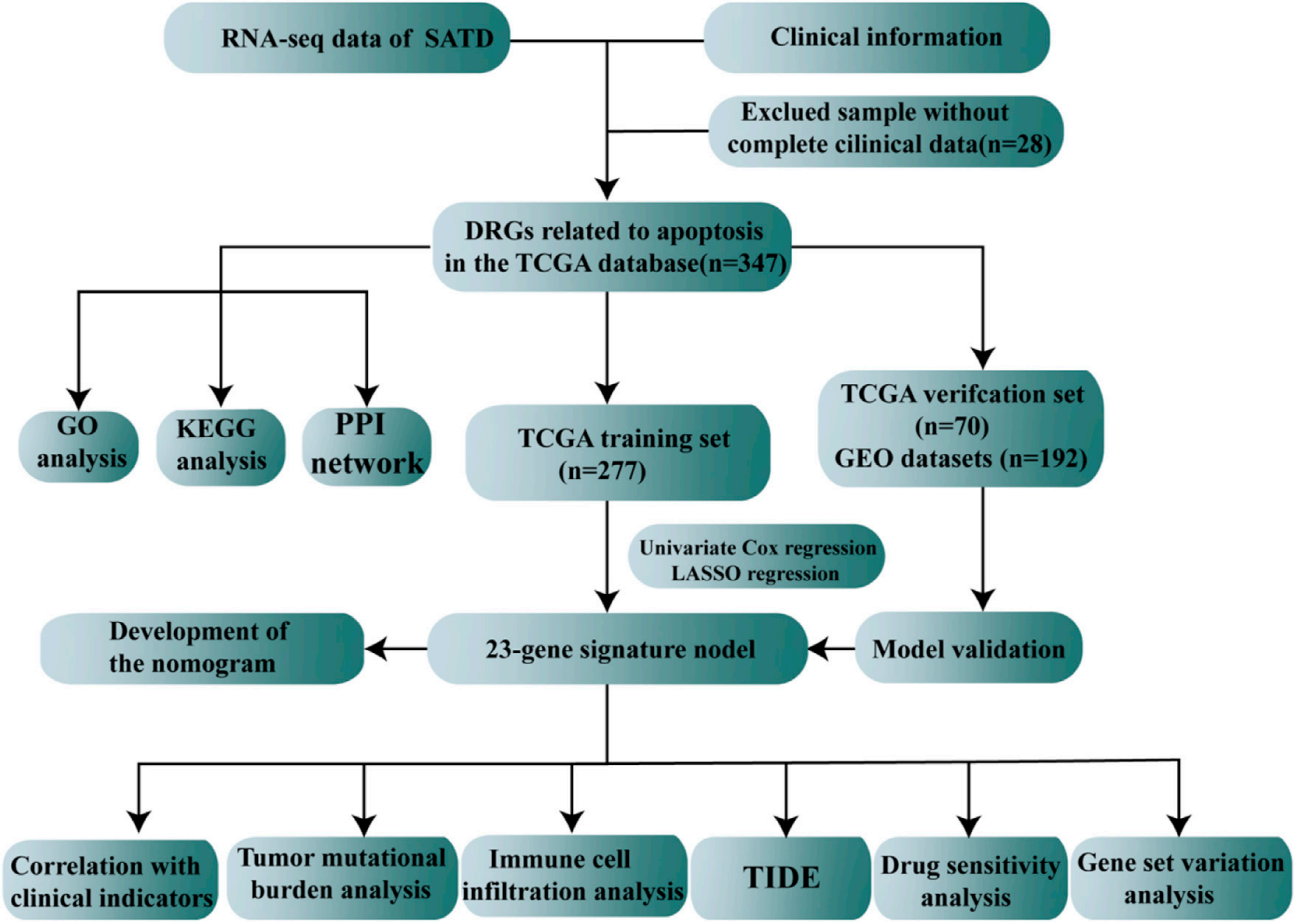
Schematic presentation of our research.

TCGA database (https://portal.gdc.cancer.gov/), the largest database of cancer gene information available, contains data, including gene expression data, miRNA expression data, copy number variants, DNA methylation and SNPs. We downloaded the STAD raw mRNA expression data (FPKM), including data for the normal (*n* = 32) and tumor groups (*n* = 375). The clinical characteristics, such as age, sex, mutation status, survival time and survival status, were extracted from the dataset. The GSE15459 dataset was obtained from the GEO database (http://www.ncbi.nlm.nih.gov/geo/), annotated on GPL570, including 192 STAD patients with complete expression profiles and survival information. A total of 3,767 apoptosis gene sets were obtained through the GeneCards database (https://www.genecards.org/). Differentially expressed genes (DEGs) were identified between tumor patients and normal patients using “limma,” and the differential apoptosis-related genes were screened with cutoffs of Padj < 0.05 and | log2FC| > 0.585. The entire process of apoptosis-related gene model construction and multi-omics data analysis were demonstrated in Figure 1.

### Gene ontology and kyoto encyclopedia of genes and genomes annotation of differentially expressed apoptosis gene sets and PPI network establishment

Functional annotation of DEGs was performed using the “ClusterProfiler” R package to fully explore the functional relevance of DEGs. Gene Ontology (GO) and the Kyoto Encyclopedia of Genes and Genomes (KEGG) analyses were performed to assess relevant functional categories. The protein–protein interaction (PPI) network of DEGs was evaluated by utilizing the Search Tool for Retrieval of Interacting Genes/Proteins (STRING, https://string-db.org) database. The PPI network was then reconstructed with Cytoscape software. Core clusters located in densely connected regions were found using the MCODE plugin, and the connectivity of network nodes was calculated.

### Establishment of a prognostic apoptosis-related gene signature

The dataset TCGA-STAD was classified into training and validation groups at a 4:1 ratio with a randomized approach. We performed univariate Cox regression survival analysis with the “Survival” package in R software. Apoptosis-related genes associated with survival were extracted for further study. Apoptosis-related genes associated with survival in apoptosis were selected for LASSO regression using the “glmnet” package to further construct prognostic correlation models. After incorporating the expression values of each specific gene, a risk score (RS) formula was explored for each sample and weighted by its estimated regression coefficient in the LASSO regression analysis. The RS of each sample was calculated using the following formula:

Risk Score (RS) = MKNK2 × (−0.224005905974604) + INCENP × (−0.128359170901958) + MSX2 × (−0.0489015408467461) + CKS2 × (−0.015445913414839) + TFF1 × 0.000656550614025462 + TTR × 0.00996710615554725 + IBSP × 0.0100666591137116 + F2R × 0.0112147264956773 + GRP × 0.0434762335766126 + FRZB × 0.0541881827853097 + APOB × 0.0552096785167862 + TUBB4A × 0.061903840751161 + ZFP36 × 0.0619726749892716 + KIT × 0.0720220065121335 + KRT7 × 0.0789272155075932 + ACKR3 × 0.083145441094113 + SNCG × 0.0852199231104383 + HBB × 0.0867586669714403 + CDC6 × 0.125436438794775 + CXCR4 × 0.145672972631166 + SYT13 × 0.184991923388196 + CAST × 0.213930687288837 + CCT6A × 0.319958370169231.

Each sample was classified into low-risk and high-risk groups with the median RS as the cutoff point, and the difference in survival between the two groups was estimated using Kaplan–Meier analysis. The role of the risk score in predicting patient prognosis was examined using LASSO regression analysis and stratified analysis. Finally, we used the “survivalROC” package in R to generate the ROC curves.

### Development of a nomogram and correlation analysis of clinical characteristics

First, we performed univariate and multivariate Cox regression analyses to establish the clinical characteristics related to prognosis. We utilized the “rms” package to explore a nomogram according to apoptosis risk scores and clinical characteristics. The clinical characteristics inclued in the nomogram were identified *via* the inverse stepwise selection of variables based on the Akaike information criterion. Calibration curves (consistency index, C-index) were used to measure the predictive accuracy of the nomograms. The apoptosis risk score and the clinical characteristics of GC were compared with survival status and survival time to determine whether the apoptosis risk score can be regarded as an independent prognostic factor. We generated box plots to analyze the correlation between the apoptosis risk score and clinical characteristics.

### Internal validation cohort and external validation cohort to validate the prediction model

We used the internal validation set TCGA-STAD and the GSE15459 dataset as the validation set, which were classified into high-risk and low-risk groups according to the median apoptosis risk score. Kaplan–Meier survival analysis was used to explore the differences between the two subgroups. To further evaluate the accuracy of the apoptosis score estimation model, the internal validation set from TCGA and the GSE15459 dataset were used to generate AUC values for 2, 3, and 5 years in R. *p* < 0.05 was considered statistically significant.

### Tumor mutational burden analysis of TCGA data in gastric cancer

In the present study, we analyzed somatic mutation data for each sample with VarScan2 and then calculated the tumor mutational burden (TMB) as the total number of somatic mutations/all bases (in units of mutations/Mb). The 30 genes with the highest mutation frequency were selected for display, and the mutation landscape was mapped for each sample with the “maftools” R package.

### Tumor immune dysfunction and exclusion immunotherapy score analysis

Tumor immune dysfunction and exclusion (TIDE) is a computational method for predicting immune checkpoint blockade response. It can predict tumor response to immunotherapy by computer prediction of scores that induce T cell dysfunction in tumors with high infiltration of cytotoxic T lymphocytes (CTL) and block T cell infiltration in tumors with low CTL levels. Based on the expression profile prior to tumor treatment, the TIDE module predicts patient response by estimating multiple published transcriptomic biomarkers. The analysis related to tumor immune dysfunction and exclusion in this study was mainly done through the TIDE website (http://tide.dfci.harvard.edu/).

### Immune cell infiltration analysis

CIBERSORT is a tool for analyzing cell composition from gene expression profiles and is the most frequently cited tool. RNA-seq data from different subgroups of STAD patients were analyzed with the CIBERSORT algorithm. We used CIBERSORT to extrapolate the relevant proportions of the 22 immune-infiltrating cells by downloading the source code of the CIBERSORT R language calculation and the LM22 signature gene matrix sequence. The relative infiltration ratios of the 22 different immune cell types in the samples were assessed using the “preprocessCore” and “e1071” R packages. Finally, Spearman correlation analysis was performed in R to assess the association between immune cell infiltration and gene expression. *p* < 0.05 was considered significantly different.

### Drug sensitivity analysis

Based on the largest pharmacogenomics database, the GDSC Cancer Drug Susceptibility Genomics Database (https://www.cancerrxgene.org/), we utilized the “pRRophetic” R package to forecast the drug sensitivity of tumor samples from both high- and low-risk groups. pRRophetic is a powerful and widely used algorithm, which was broadly adopted in medical research (Liu et al., 2021; Liu et al. 2022b; Liu et al. 2022c). Regressions were applied to obtain IC50 estimates for specific chemotherapeutic drug treatments, and 10 cross-validations were used to measure the accuracy of the regressions and predictions with the GDSC training set. Default values were selected for all parameters, including “battle” to remove batch effects and repeat gene expression averages.

### Gene set variation analysis between the high- and low-risk groups

Gene set variation analysis (GSVA) is a nonparametric unsupervised method to assess the enrichment in transcriptomic gene sets (Huang et al., 2017). GSVA converts gene-level changes into pathway-level changes by synthetically scoring the genome of interest to identify the biofunctions of the samples. In the present study, we downloaded gene sets from the Molecular Signatures Database (v7.0), and we used the “GSVA” R language package and the GSVA algorithm to comprehensively score each gene set to assess the potential biological function changes of different samples.

### Statistical analysis

The survival curves in the present study were plotted by the Kaplan–Meier method and tested by log-rank analysis. Multivariate analysis was used for the Cox proportional risk model. ROC curve was generated by “survivalROC” package in R. C-index were used to measure the predictive accuracy of the nomograms and compared by Hosmer-Lemeshow test. The differences between the clinical characteristics and the apoptosis risk score were measured by Student’s *t*-tests or Kruskal-Wallis tests. Dysfunction socre, exclusion score and tumor mutations were measured by Wilcoxon tests. Spearman correlation analysis was performed in R to assess the association between immune cell infiltration and gene expression. All statistical analyses were performed using R language (version 4.0). All statistical tests were two-sided, and *p* < 0.05 was considered statistically significant.

## Results

### Investigation of the expression of DRGs related to apoptosis in the STAD cohort

**FIGURE 2:**
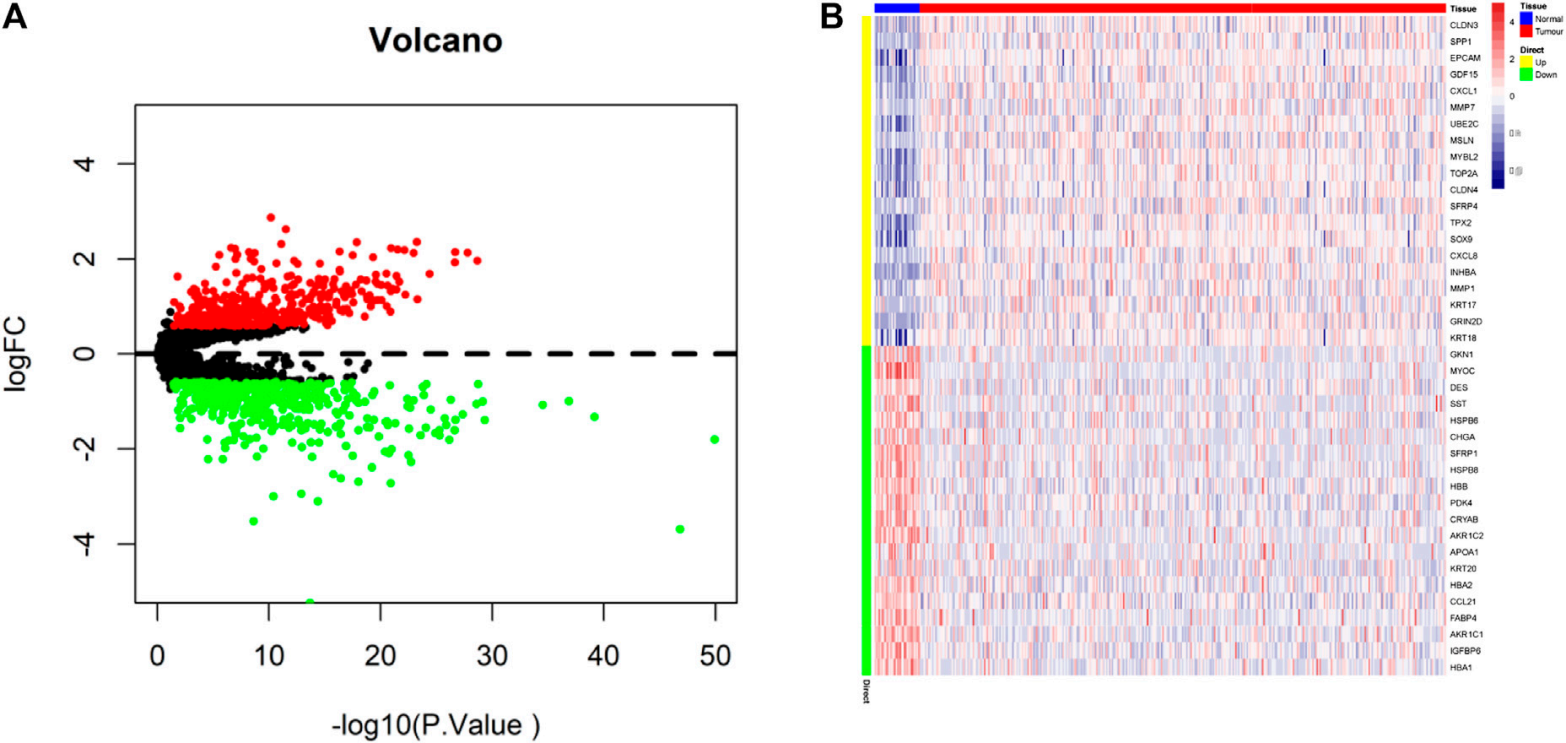
Identification of differentially expressed apoptotic genes. **(A)** Volcano map of differentially expressed apoptotic genes (Upregulated genes in red, downregulated genes in green). **(B)** TCGA-STAD of 20 up and downregulated differentially expressed apoptotic genes between tumor and normal tissues.

We extracted the raw mRNA expression data (FPKM) of processed STAD from TCGA database and obtained a total gene set of 3,767 apoptotic genes through the GeneCards database (https://www.genecards.org/). Differential analysis was performed with the “limma” package, which obtained 839 DEGs (Supplementary Table S1 and Figure 2A) for subsequent analysis with 406 upregulated genes (Supplementary Table S2) and 433 downregulated genes (Supplementary Table S3). The heatmap in Figure 2B shows the top 20 up and downregulated genes.

### Functional enrichment of differential genes and construction of protein interaction networks

**FIGURE 3:**
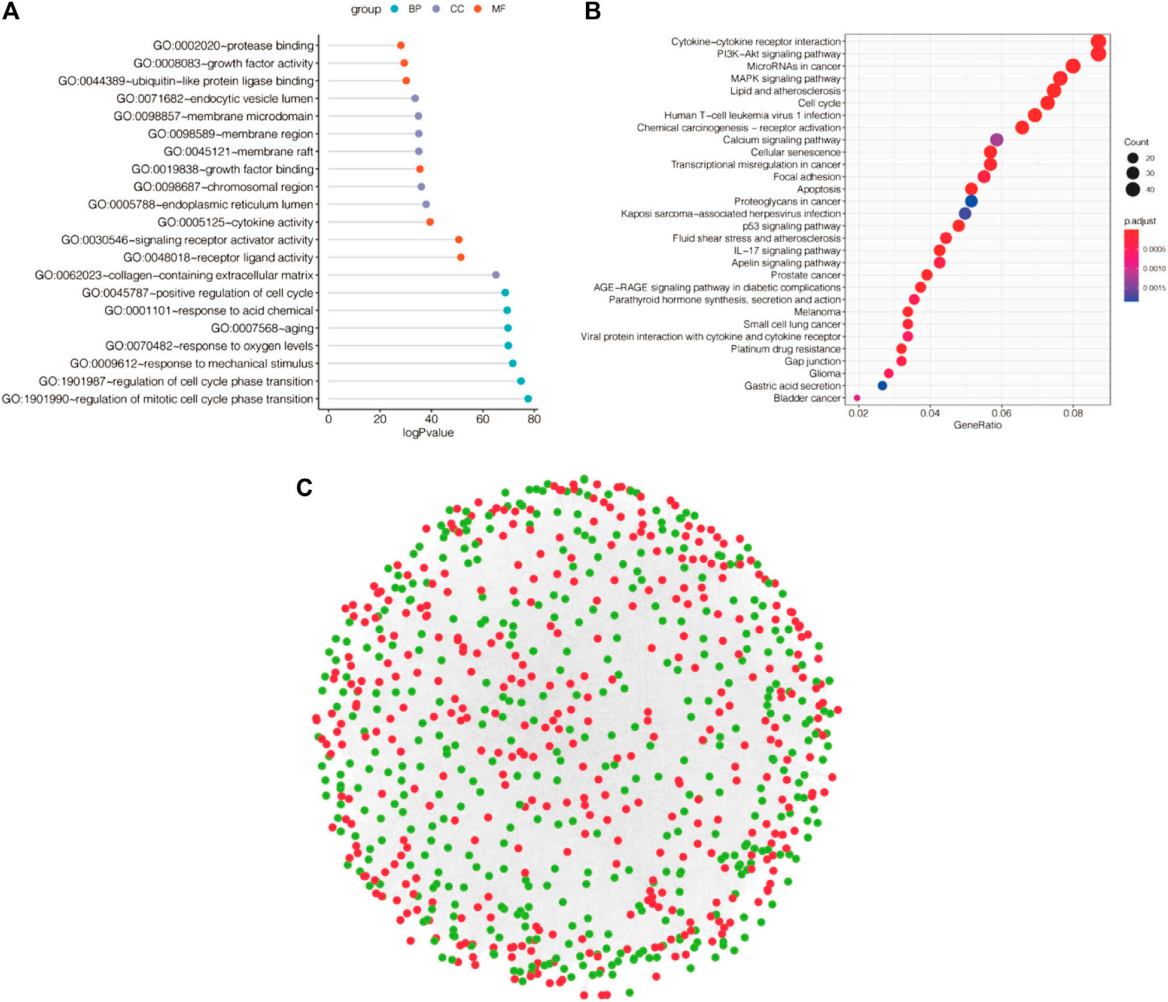
Functional enrichment analysis and PPI networks for differentially expressed genes. **(A)** GO enrichment analysis of differentially expressed apoptotic genes. **(B)** KEGG enrichment analysis of differentially expressed genes. **(C)** PPI network. (Upregulated genes in red, downregulated genes in green).

We further performed pathway analysis of DEGs related to apoptosis, and the GO results showed that these DEGs to be mainly enriched in the regulation of mitotic cell cycle phase transition, regulation of cell cycle phase transition and response to mechanical stimulus (Figure 3A). Based on KEGG analysis showed that the DEGs were mainly enriched in cytokine–cytokine receptor interactions, the PI3K-Akt signaling pathway, microRNAs in cancer and other pathways (Figure 3B). We utilized Cytoscape software to conduct PPT network analysis using the DEGs (upregulated genes are shown in red, and downregulated genes in green in Figure 3C).

### Identification of prognosis-related genes and construction of predictive models

**FIGURE 4:**
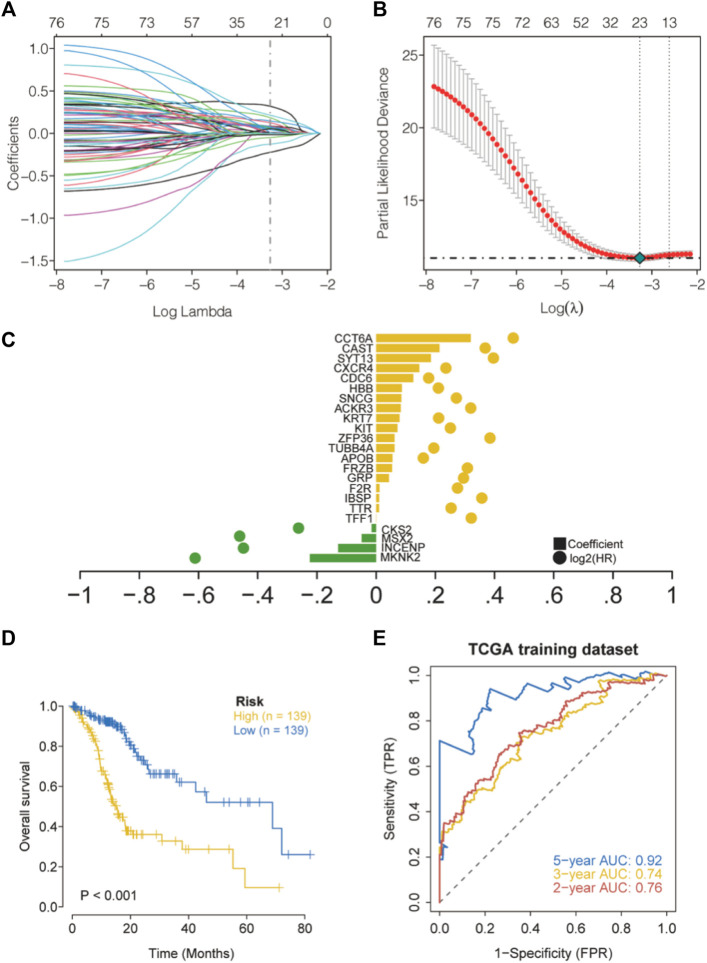
Construction of apoptosis risk scores in the cohort TCGA-STAD. **(A)** LASSO coefficient curves for 77 prognostic apoptotic genes in the training set TCGA-STAD. **(B)** The coefficient profile plot was generated with log (*λ*). **(C)** The regression coefficients and log2 (HR) for 23 genes associated with apoptosis. **(D)** Kaplan–Meier curves for high- and low-risk groups. **(E)** Validation of ROC curves for the apoptosis risk score in the training dataset from TCGA.

First, univariate Cox regression models were applied based on apoptotic DEGs in the training set from TCGA, and a total of 77 apoptosis-related genes were found to be significantly associated with overall survival (OS) (Supplementary Table S4). LASSO regression analysis was then performed on these 77 apoptotic genes to calculate the correlation coefficients. We used the LASSO algorithm for the training cohort from TCGA to further identify the 23 best candidate genes to generate apoptosis risk scores (Figures 4A–C). The coefficients for these 23 genes are shown in Supplementary Table S5. In the training cohort, patients were categorized into low-risk and high-risk groups according to the apoptosis risk score. Notably, Kaplan–Meier curves indicated that patients in the low-risk group had longer OS than patients in the high-risk group (Figure 4D). The accuracy of the apoptosis risk scores was reflected by the AUC values for 2-, 3- and 5-years OS, which were 0.76, 0.74, and 0.92, respectively (Figure 4E). The training set AUC values all suggested that the model had good validation efficacy.

### Development of the nomogram and correlation analysis of multiple clinical indicators

**FIGURE 5:**
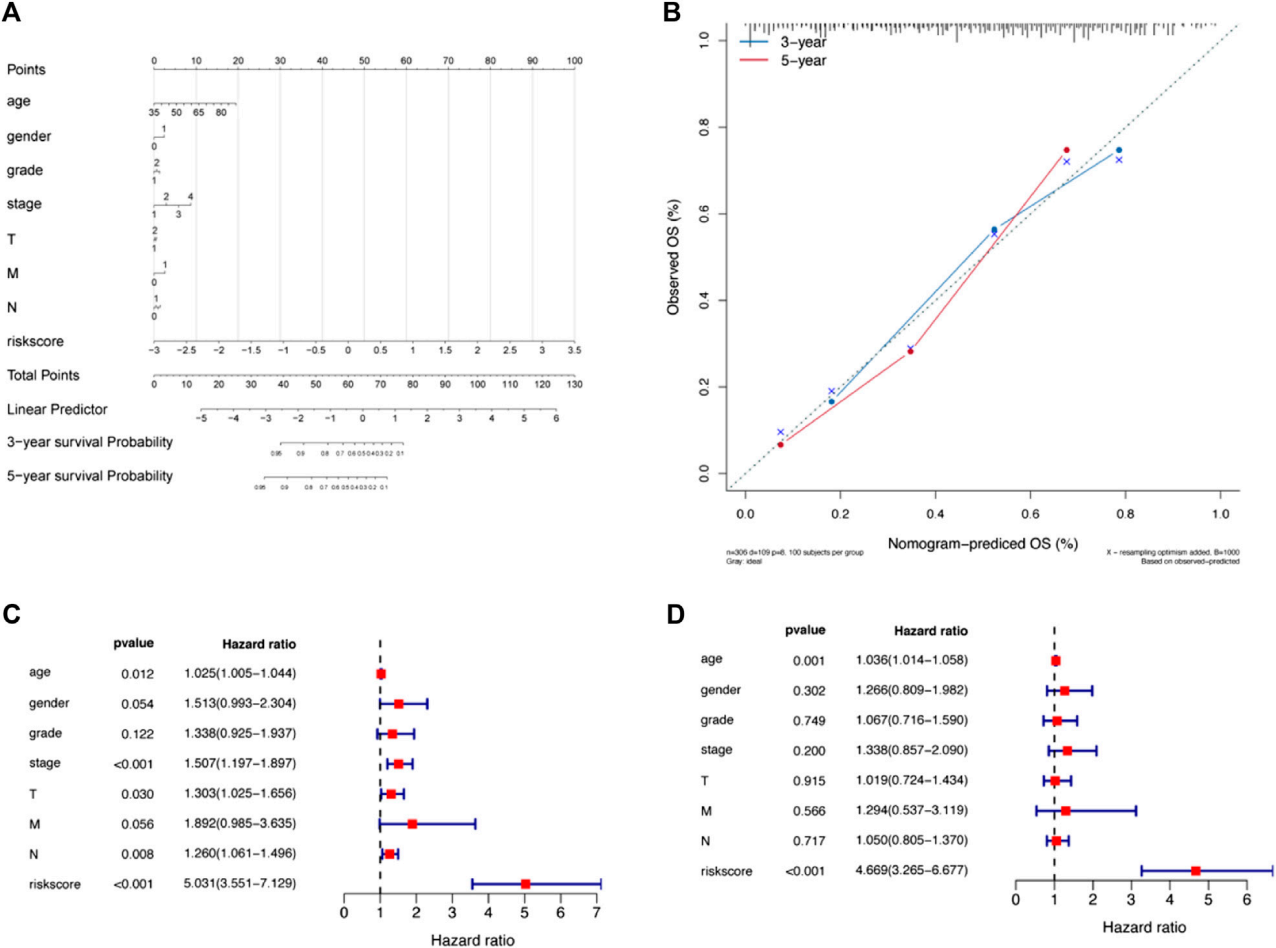
A nomogram was constructed combining apoptosis risk scores and clinicopathological characteristics in the cohort from TCGA-STAD. **(A)** A nomogram predicting 3- and 5-years overall survival was constructed. **(B)** Calibration curves of the nomogram. **(C)** Results of univariate Cox analysis of the cohort from TCGA-STAD. **(D)** Results of multivariate Cox analysis for the cohort from TCGA-STAD.

**FIGURE 6:**
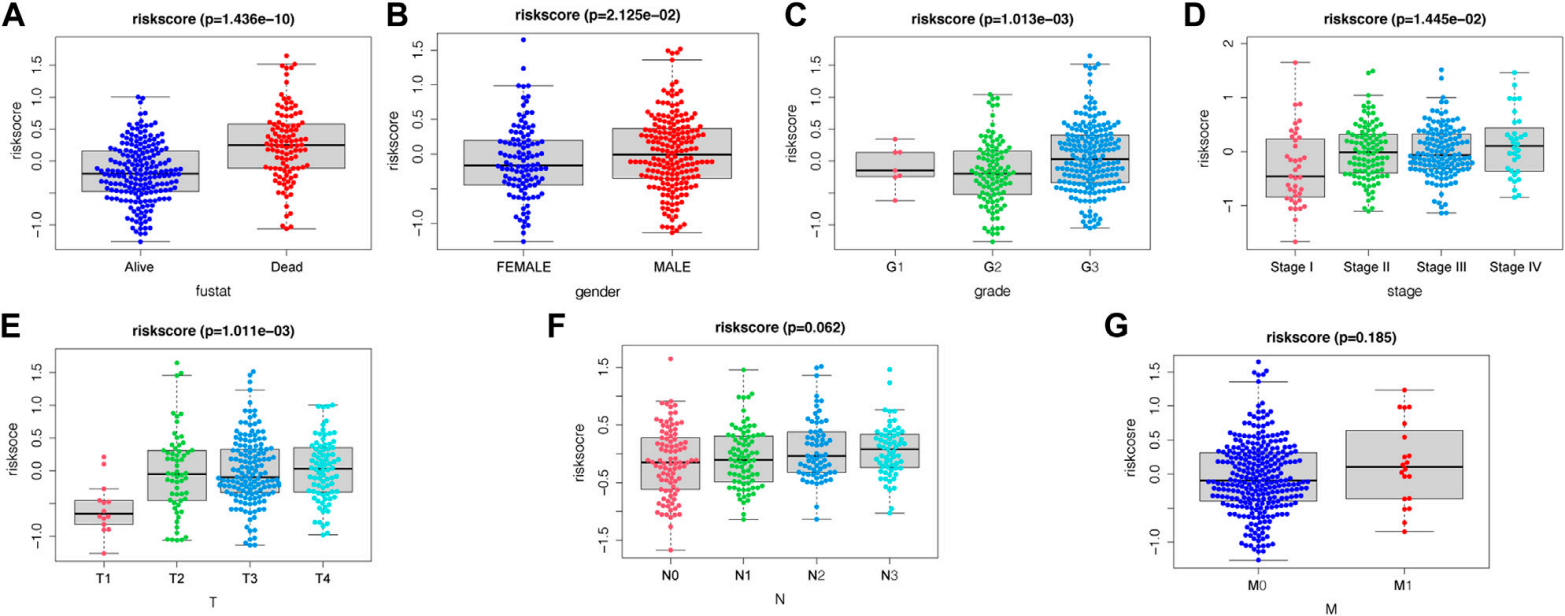
Association between apoptosis risk scores and clinical characteristics, including **(A)** survival status, **(B)** gender, **(C)** grade, **(D)** stage, **(E)** T-stage, **(F)** N-stage, and **(G)** M-stage.

In the present study, a nomogram model was constructed to predict patient prognosis. The results showed that the distribution of the values of different clinical indicators and risk score values of GC in all our samples had different degrees of contribution throughout the scoring process (Figure 5A). In addition, predictive analysis was also performed for OS at both 3 and 5 years, which indicated that the nomogram had good predictive power (Figure 5B). The risk score was found to be an independent prognostic factor for TCGA-STAD patients by univariate (Figure 5C) and multifactorial analyses (Figure 5D). Finally, we divided the samples corresponding to the risk score values into different groups, and the results of each clinical indicator are presented in the form of box line plots in Figures 6A–G. We found that the distribution of risk score values differed significantly between the groups with regard to the clinical indicators of survival status, sex, grade, stage, and T stage (*p* < 0.05).

### Verification of the apoptosis-linked gene biosignature

**FIGURE 7:**
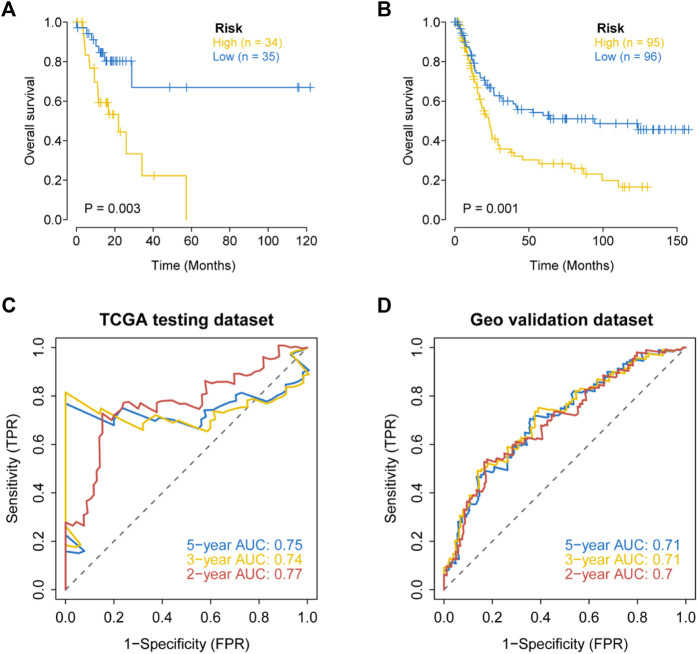
External validation of the apoptosis related genes signature in the TCGA testing dataset and the GEO dataset (GSE15459). **(A)** Kaplan–Meier curves for the OS of patients in different risk groups of the TCGA testing dataset. **(B)** Kaplan–Meier curves for the OS of patients in different risk groups of the GEO dataset (GSE15459). **(C)** Validation of ROC curves for apoptosis risk score in the TCGA testing dataset. **(D)** Validation of ROC curves for apoptosis risk score in the GEO dataset (GSE15459).

We further evaluated the prognostic value of the apoptosis risk assessment using two validation sets (TCGA internal validation set and GSE15459 dataset). The apoptosis risk score was calculated for each patient, and patients in the two validation sets were divided into high- and low-risk groups based on the median risk score. Based on the survival analysis of the two validation sets, the survival rate was lower in the high-risk group and higher in the low-risk group (Figures 7A,B). The AUC values of the ROC curves for the 2-, 3- and 5-years survival rates were 0.7, 0.71, and 0.71 for the internal validation set from TCGA and 0.77, 0.74, and 0.75 for the GSE15459 dataset, respectively (Figures 7C,D). Based on the apoptosis risk score, the prognostic model had high accuracy and stability in disease prognosis assessment. These data suggested that our prognostic risk model accurately estimates the prognosis of GC individuals with GC.

### Multiomics study to investigate the clinical predictive value of the model

**FIGURE 8:**
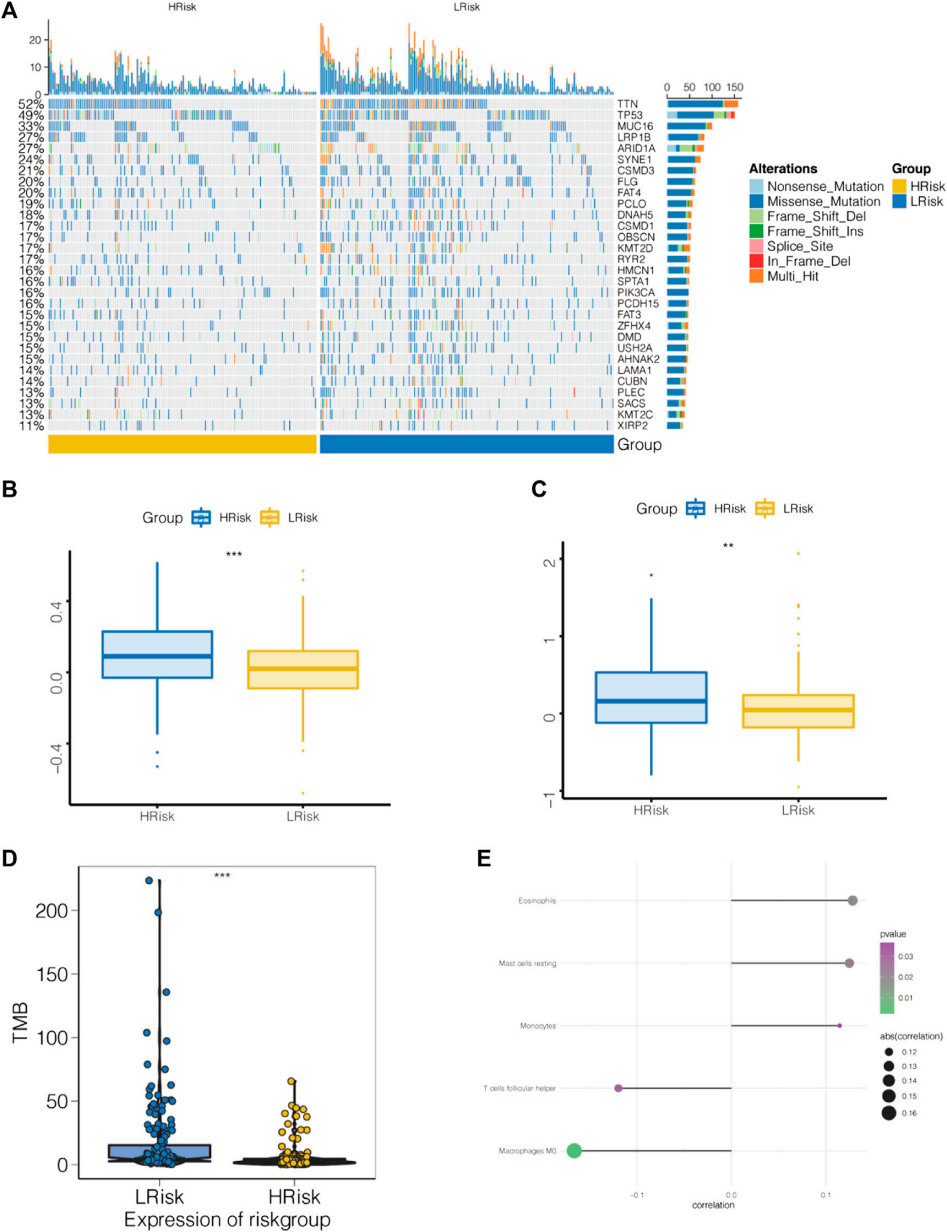
Association of apoptosis risk score with immunotherapy response and tumor mutation load. **(A)** Top 30 mutated tumor genes. **(B)** Boxplot representation of T cell dysfunction scores in the high-risk group versus low-risk group. **(C)** Boxplot representation of T cell exclusion scores in the high-risk group versus low-risk group. **(D)** Relationship between low-risk score and high-risk score tumor mutations. **(E)** Comparison of immune cell components of low-risk score and high-risk score patients by CIBERSORT. *p < 0.05, **p < 0.01, and ***p < 0.001.

The mutation profiles of patients in the high- and low-risk groups showed that the proportion of mutations in genes, such as TTN, was significantly lower in the high-risk group than in the low-risk group (Figure 8A). In the STAD-TCGA cohort, compared with GC patients with high-risk score, low-risk socre with GC patients got a lower T cell exclusion score and T cell dysfunction score (Figures 8B,C). We also found a significantly lower TMB in the high-risk group (Figure 8D).

The tumor microenvironment (TME) is mainly composed of tumor-associated fibroblasts, immune cells, the extracellular matrix, multiple growth factors and inflammatory factors. The TME significantly influences the survival outcome and clinical treatment response of tumor patients. We further explored the potential molecular mechanisms by which the apoptosis risk score affects GC by analyzing the relationship between the apoptosis risk score and tumor immune infiltration. This analysis revealed a significantly higher proportion of eosinophils, resting mast cells and monocytes as well as a lower proportion of M0 macrophages and follicular helper T cells in the high-risk patient group than in the low-risk patient group (Figure 8E).

**FIGURE 9:**
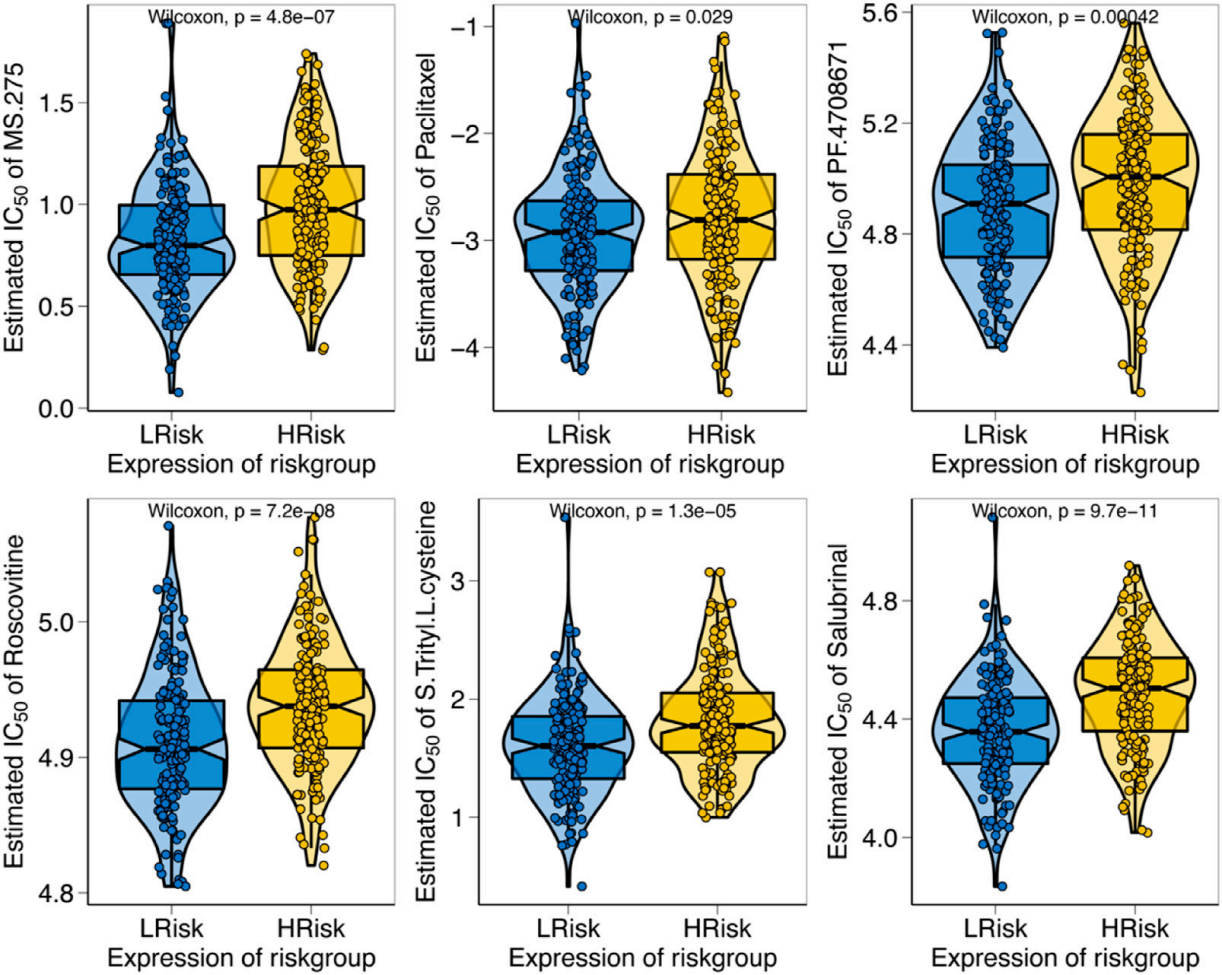
Relationship between apoptosis risk score and chemotherapy resistance. The figure illustrates the correlation between the apoptosis risk score and the IC_50_ of various drugs in patients with GC.

We predicted the chemotherapy sensitivity of each tumor sample in TCGA-STAD using the drug sensitivity data from the GDSC database with the “pRRophetic” R package, and the relationship between the apoptosis risk score and sensitivity to chemotherapeutic drugs was further explored. According to the results demonstrated that the apoptosis risk score significantly influenced the sensitivity of patients to MS.275, paclitaxel, PF.4708671, roscovitine, S-trityl-L-cysteine, and salubrinal. Patients with GC in the high-risk group had a worse response due to a higher IC50 (Figure 9).

### Prognostic modeling with GSVA to explore specific signaling mechanisms

**FIGURE 10:**
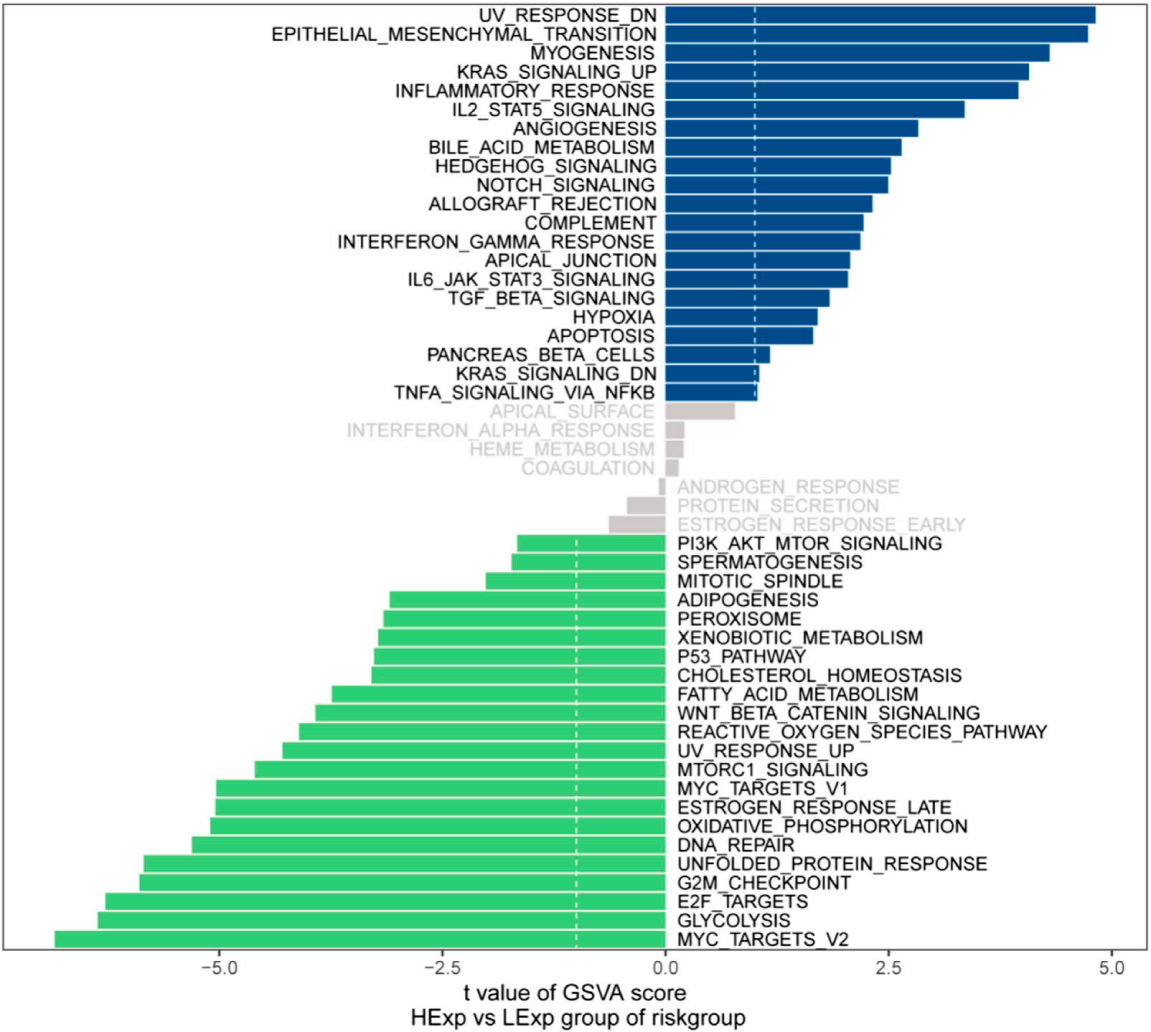
Differences in GSVA enrichment among apoptosis risk score groups.

We next investigated the specific signaling pathways involved in the models associated with the high- and low-risk groups to explore the potential molecular mechanisms by which risk scores affect tumor progression. The GSVA results showed that the differential pathways in the two groups were mainly enriched in the UV_RESPONSE_DN, EPITHELIAL_MESENCHYMAL_TRANSITION, MYC_TARGETS_V2 and GLYCOLYSIS signaling pathways (Figure 10). These results indicate that these signaling pathways may affect the prognosis of high- and low-risk GC patients.

## Discussion

GC has a high degree of malignancy (Bray et al., 2018). Despite years of research focused on its basic and clinical aspects and numerous clinical trials to evaluate new treatments, the prognosis for patients with GC remains unsatisfactory. Patients diagnosed with GC undergo various treatments, such as surgery, biologically targeted therapy and chemotherapy, with unsatisfactory survival rates (Duan et al., 2019). Therefore, it is important to classify the various patients to facilitate access to effective treatment. Apoptosis is a programmed form of cell death that does not provoke an inflammatory response. Such silent cell death can be triggered by DNA damage, cytochrome C release or the tumor necrosis factor (TNF) receptor superfamily activation (Beroske et al., 2021). Disorders of apoptosis can result in a number of diseases, such as immune diseases or cancers. An important feature of cancer is that cells lose their apoptotic characteristics and proliferate indefinitely (Hanahan and Weinberg 2011). Overall, there is increasing interest in using multiomics analysis to assess tumor prognosis in cancer, including breast, lung, gastric and colorectal cancers (Li et al., 2016; Xiao et al., 2019; Zhang et al., 2020b; Yuan et al., 2021). However, no study to data has reported the multiomics analysis of apoptosis-related genes in gastric cancer. Therefore, we combined apoptosis-related genes to construct a model for the prognostic assessment of GC. First, we identified 839 apoptosis-related DEGs in GC, 77 of which were correlated with prognosis. Second, we developed and validated a novel apoptosis risk score by integrating TCGA-STAD and GSE15459 cohorts. In addition, we found that the apoptosis risk score predicts the clinical characteristics, TMB, TME and chemotherapeutic drug sensitivity in GC.

The present study demonstrated a generally higher TMB in the low-risk group and lower in the high-risk group. Related studies have shown that tumors with a higher TMB have increased DNA mutations, which results in a greater production of candidate peptides, leading to a stronger likelihood of successful recognition of neoantigens by the immune system and a better prognosis for tumor patients (Ritterhouse 2019). Recently, Yang et al. (2020) showed that TTN mutations in GC patients are associated with a higher TMB and better prognosis of patients. Similarly, Chen et al. (2021b) repotorted that EP300-mutated cancers have an increased TMB, antitumor immunoreactivity and PD-L1 expression. In addition, Schrock et al. (2019) found that TMB is an important independent biomarker in MSI-H mCRC. In mCRC, TMB can be used to stratify patients to determine the probability of response to immunotherapy. Thus, TMB has emerged as a potential predictive biological marker for the treatment of various tumors. T cells play an important role in tumor immunity. Related studies have revealed the mechanism of T cell immune evasion in tumors: In some tumors, cytotoxic T cells have a high degree of infiltration, but they are in a dysfunctional stat; immunosuppressive factors can remove T cells infiltrating in tumor tissue in other tumors (Spranger and Gajewski 2016). Apoptosis-related genes have an important effect on T cell tumor immunity. For example, p53 promotes T cell homeostasis by inducing pro-apoptotic SAP (Madapura et al., 2012). TIDE scores can show Dysfunction and Exclusion of T cells. Our study shows that low-risk patients have lower T cell Dysfunction and Exclusion compared to high-scoring patients.

The TME in solid tumors is heterogeneous and is characterized by immune cells, as a key component stromal cells, blood vessels and the extracellular matrix (ECM) as its hallmark features (Vaillant et al., 2021). A key component of the TME is immune cells. Tumor infiltration by different adaptive and innate immune cells leads to different effects, including promotion or inhibition of tumor growth (Anderson and Simon 2020). Apoptosis also plays an important role in TME. For example, BCL-2 (B cell lymphoma-2), one of the most valued genes in apoptosis research, has a significant inhibitory effect on apoptosis. BCL-2 in T cells induces immunosuppression by enhancing Treg abundance and CTL depletion (Liu et al., 2022a). Tumor cell apoptosis has been the focus of tumor research, but the potential regulatory role between apoptosis and immunity is still not fully elucidated. As based on our apoptosis-related risk score, the present study revealed that apoptosis may be highly correlated with tumor immunity. GC patients with higher risk scores had a higher proportion of monocytes and resting mast cells. Increased infarction of monocyte and mast cell counts is an important predictor of poor clinical outcome in a variety of tumors, such as GC, colorectal cancer and pancreatic cancer (Fleischmann et al., 2009; Feng et al., 2018; Yamamoto et al., 2020). Monocytes are involved in various tumor stages, such as in the initial stages of tumor growth and in distant metastases (Olingy et al., 2019). Ribatti et al. (2010) revealed that mast cell density is significantly associated with angiogenesis and progression in patients with GC. CCL2 is a major chemokine recruited by monocytes in a mouse model of colitis-associated colorectal cancer, and CCL2 expression increases with tumor progression (Chun et al., 2015). Mast cells are an important component of solid tumors and play different roles in the TME. *In vitro* experiments have demonstrated that tumor-derived adrenomedullin (ADM) induces hypertrophic release of interleukin 17A through the PI3K-AKT signaling pathway, promoting proliferation and inhibiting apoptosis of GC cells (Lv et al., 2018). Interestingly, a high mast cell density inhibits the growth of prostate cancer, and mast cell density is associated with a good prognosis (Fleischmann et al., 2009).

Chemotherapy is one of the most significant treatments for progressive GC. Apoptosis is a vital molecule mechanism targeted by antineoplastic therapy (Yu and Silva 2018). Such as, celastrol triggers apoptosis by means of targeting of peroxidase-2 (Chen et al., 2020). Apatinib induces apoptosis by targeting the PI3K/Akt signaling pathway (Jia et al., 2019). However, different patients have various responses to antineoplastic drugs. Recently, precision medicine has sparked an interest in cancer drug sensitivity prediction models (Tsimberidou et al., 2020), and the use of computational methods for drug sensitivity prediction has received increasing attention (Sun and Hu 2018; Ahmadi Moughari and Eslahchi 2021; Pouryahya et al., 2022). Furthermore, the advent of pharmacogenomic datasets has further promoted the development of prediction models. Nonetheless, accurate prediction of drug sensitivity in individuals is a challenging task for precision medicine. Precision therapy excludes the concept of one-size-fits-all and instead emphasizes the customization of an appropriate drug for each individual. The main concept of precision therapy is to recommend drugs according to the genomic profile of a patient. The present study found that the apoptosis risk score significantly impacted the sensitivity of GC patients to MS.275, paclitaxel, PF.4708671, roscovitine, S-trityl-L-cysteine, and salubrinal. Although MS.275, PF.4708671, roscovitine, S-Trityl-L-cysteine and salubrinal are not utilized as chemotherapy of GC, elaboration of the association between apoptosis risk scores and drug sensitivity may reveal a potential therapeutic role for these agents. Our molecular stratification of GC patients based on apoptosis scores to predict drug sensitivity may optimize oncologic chemotherapy for precision treatment.

In the previous 2 years, there have been numerous studies on signatures associated with the prognosis of GC, such as hypoxia-immune-based gene signatures (Liu et al., 2020), long non-coding RNA signatures (Ghafouri-Fard and Taheri 2020), ferroptosis-related lncRNAs signatures (Chen et al., 2021a) and immune-related signatures (Zhao et al., 2021). Although escaping programmed cell death (apoptosis) is a hallmark of tumors (Hanahan and Weinberg 2000), there are no relevant studies on the prognosis of apoptosis-related signatures in GC. Therefore, we established an apoptosis risk model with validation in internal and external validation sets. In addition, we combined a multiomics approach to explore the risk score, including drug sensitivity, TMB, TME, and TIDE score. The present study had several limitations. First, although we validated the apoptosis risk model with internal and external data, the reliability of our model needs to be confirmed in more clinical studies before it can be generalized. Second, although the prognosis of GC is unpredictable with high heterogeneity, and we only used apoptosis-related genes to construct the prognostic model, and other important key genes may be excluded. Regardless, our apoptosis risk assessment model has the potential to serve as a prognostic biomarker, providing new insights for further research and treatment of GC.

## Conclusion

We constructed and validated a novel apoptosis risk assessment model for GC consisting of 23 apoptotic genes. The model categorized GC patients into two subgroups according to the apoptosis risk score (high- and low-risk groups). Moreover, we evaluated the relationship of the apoptosis risk score with clinical features, tumor immunity, TMB, drug sensitivity and TIDE scores in GC, and demonstrated that the apoptosis risk score is a good predictor of these indicators. Thus, the apoptosis score may function as a potential molecular biological indicator for the prognosis of GC and provide new insights into the clinical prognosis and treatment of GC.

## Data Availability

The original contributions presented in the study are included in the article/Supplementary Material, further inquiries can be directed to the corresponding authors.

## Supplementary material

SUPPLEMENTARY TABLE S1 Eight hundred and thirty-nine apoptotic differentially expressed genes.

SUPPLEMENTARY TABLE S2 Four hundred and six upregulated genes.

SUPPLEMENTARY TABLE S3 Four hundred and thirty-three downregulated genes.

SUPPLEMENTARY TABLE S4 Seventy-seven apoptosis-associated genes with differential overall survival.

SUPPLEMENTARY TABLE S5 Coefficients of 23 genes.
