# Supplementary figures and images for: Application of apoptosis-related genes in a multiomics-related prognostic model study of gastric cancer
Source: Front Genet. 2022 Aug 5;13:901200. doi: 10.3389/fgene.2022.901200 (PMC9389051; doi:10.3389/fgene.2022.901200)

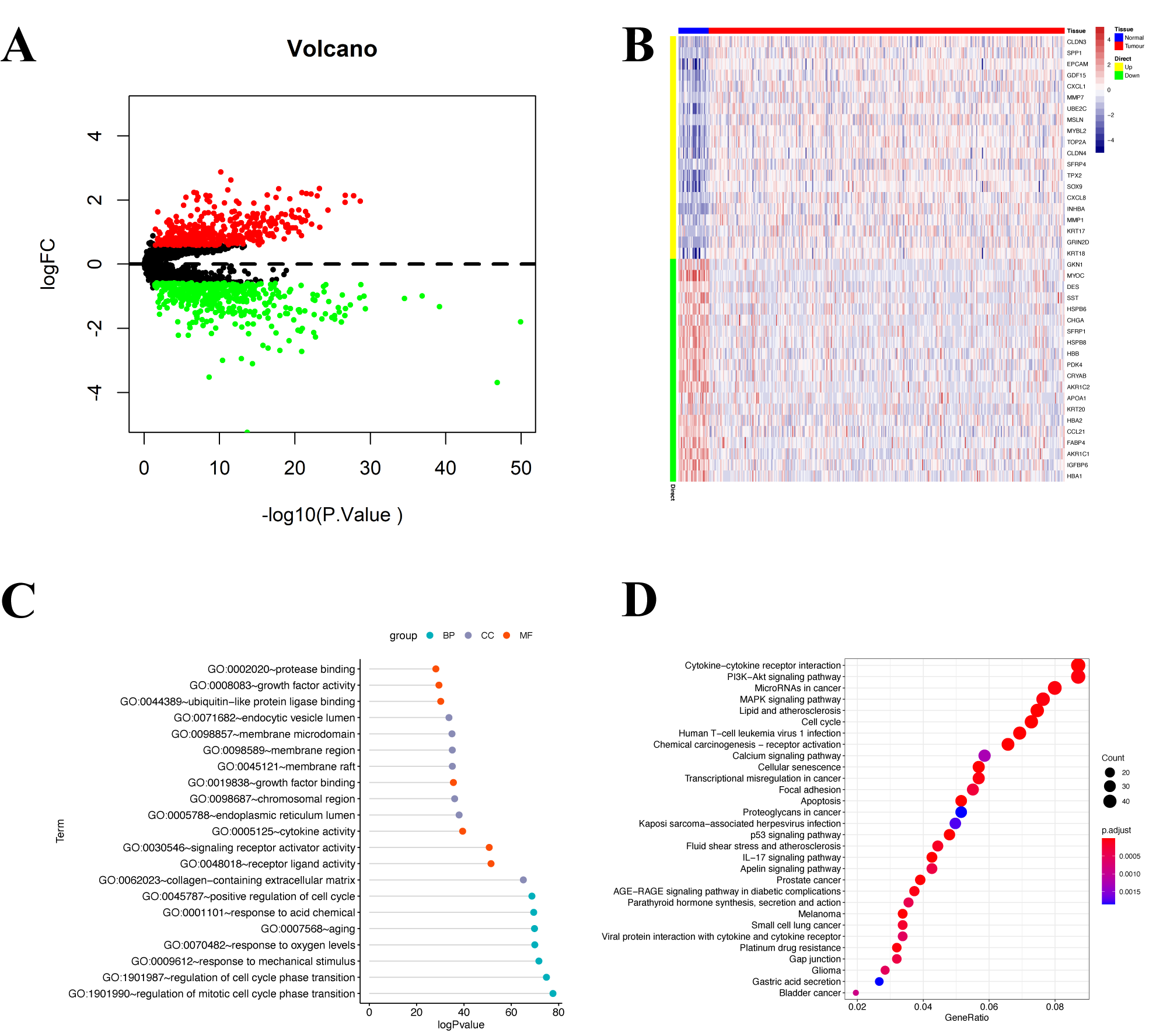

Supplement: Supplementary file 1 [file DataSheet1.ZIP › Fig 1.tif]

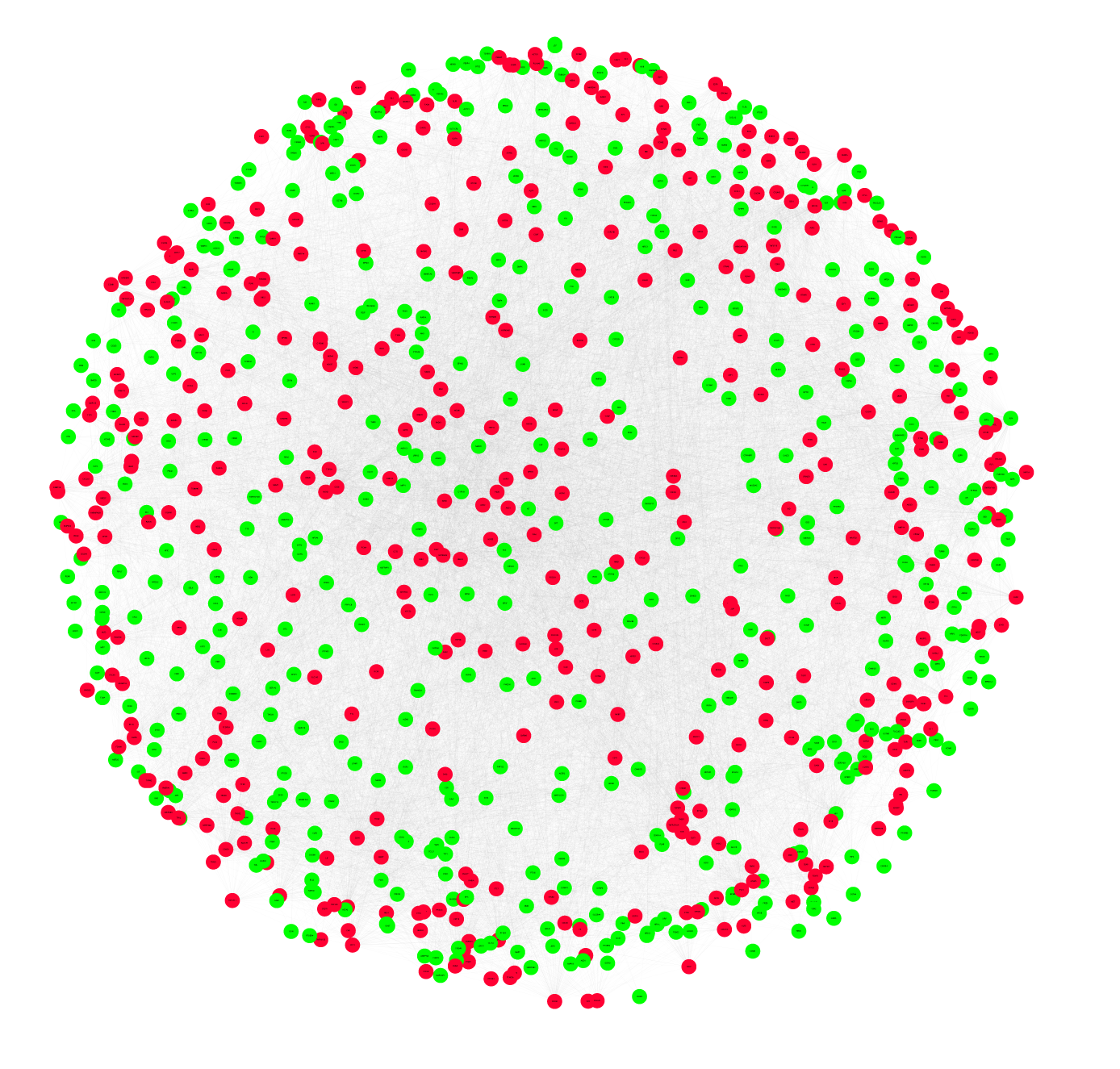

Supplement: Supplementary file 1 [file DataSheet1.ZIP › Fig2.tif]

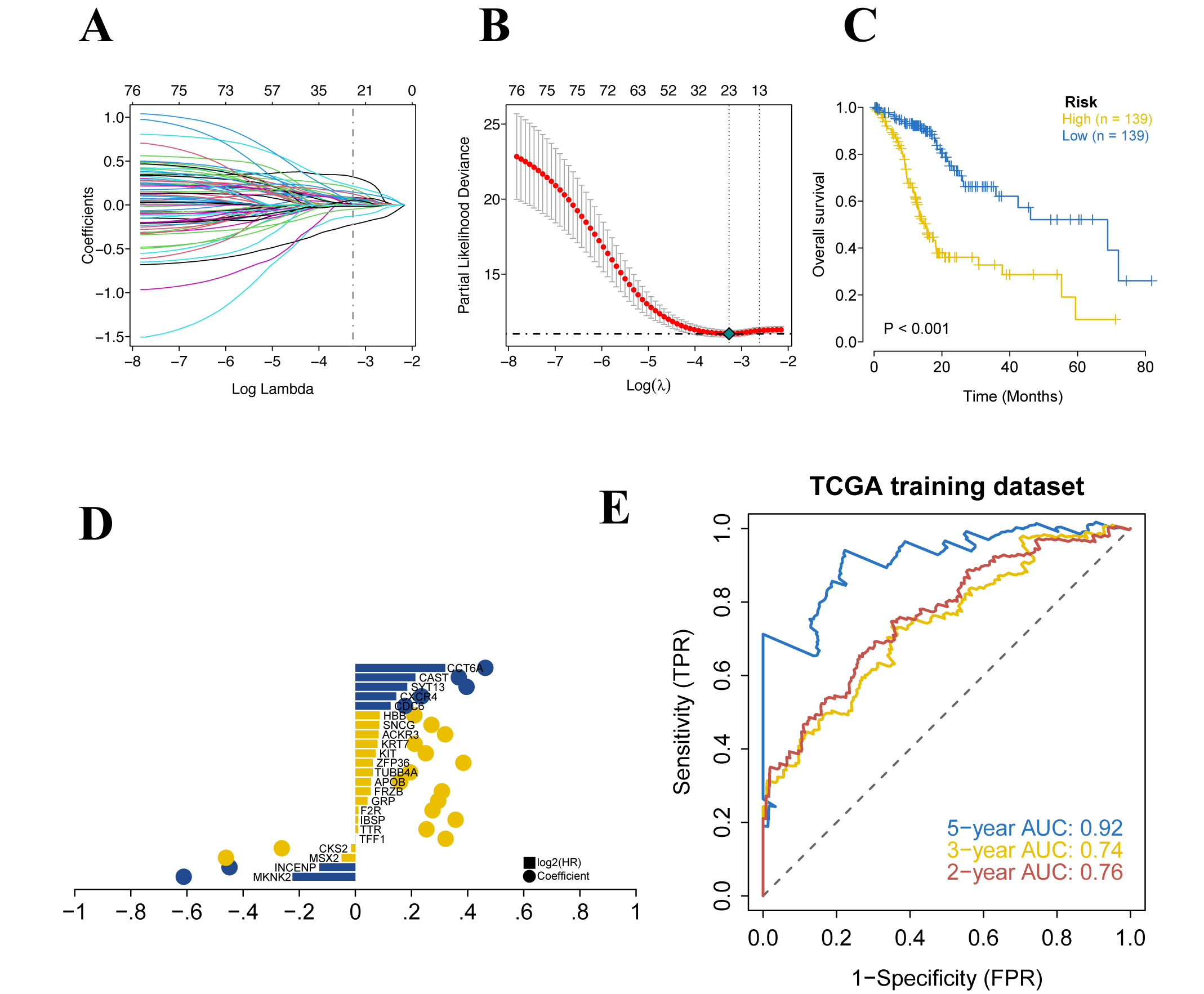

Supplement: Supplementary file 1 [file DataSheet1.ZIP › Fig3.tif]

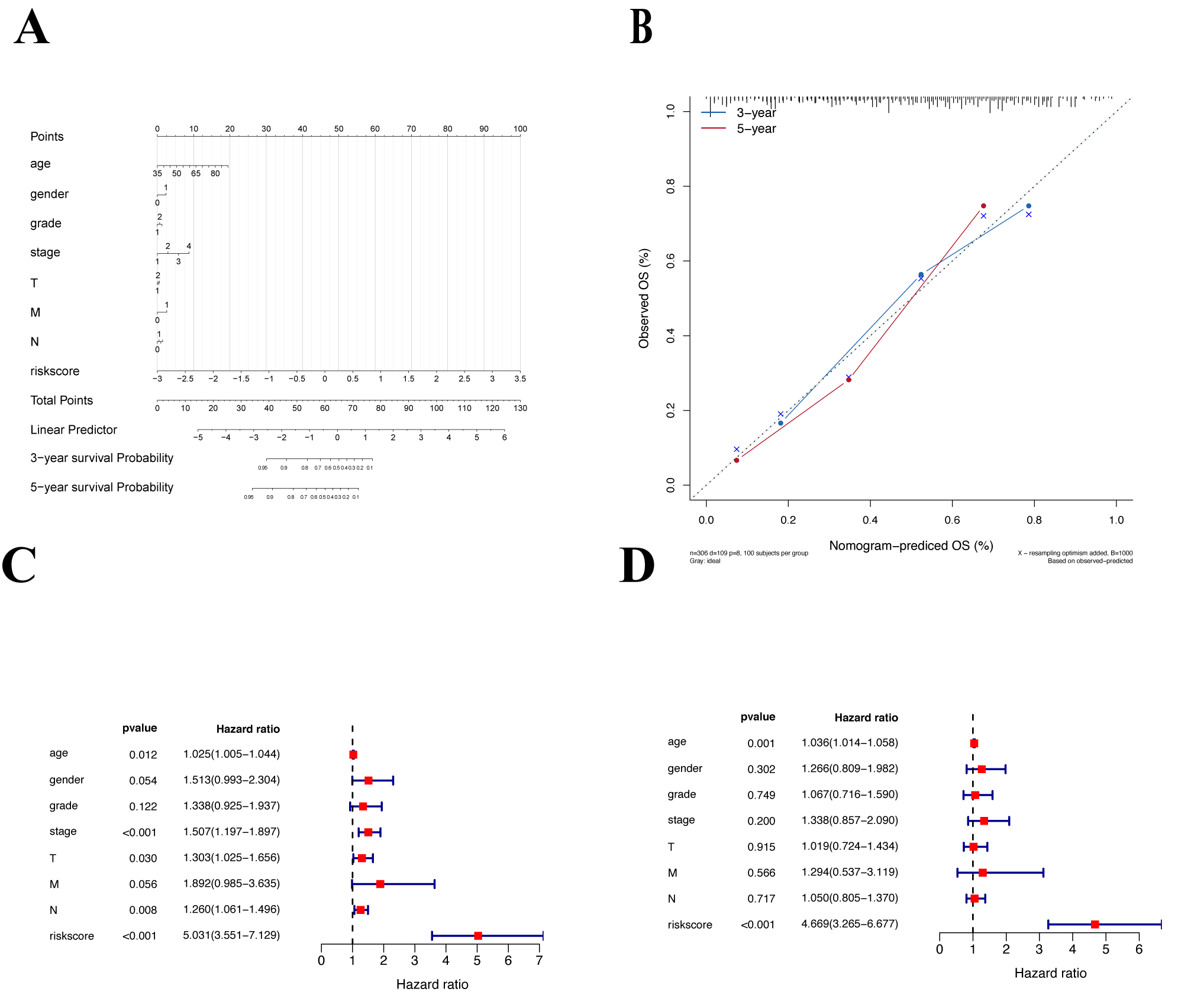

Supplement: Supplementary file 1 [file DataSheet1.ZIP › Fig4.tif]

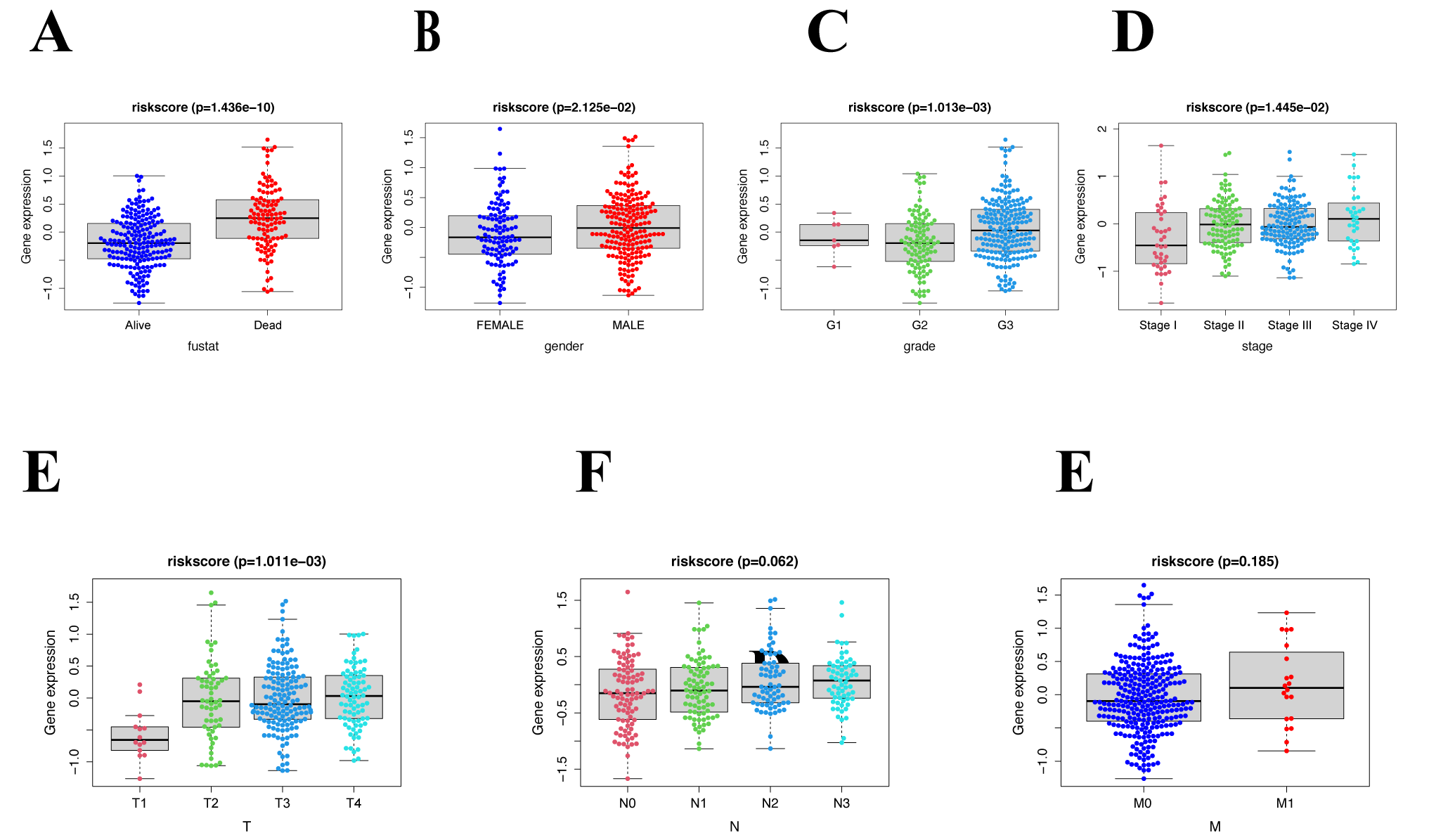

Supplement: Supplementary file 1 [file DataSheet1.ZIP › Fig5.tif]

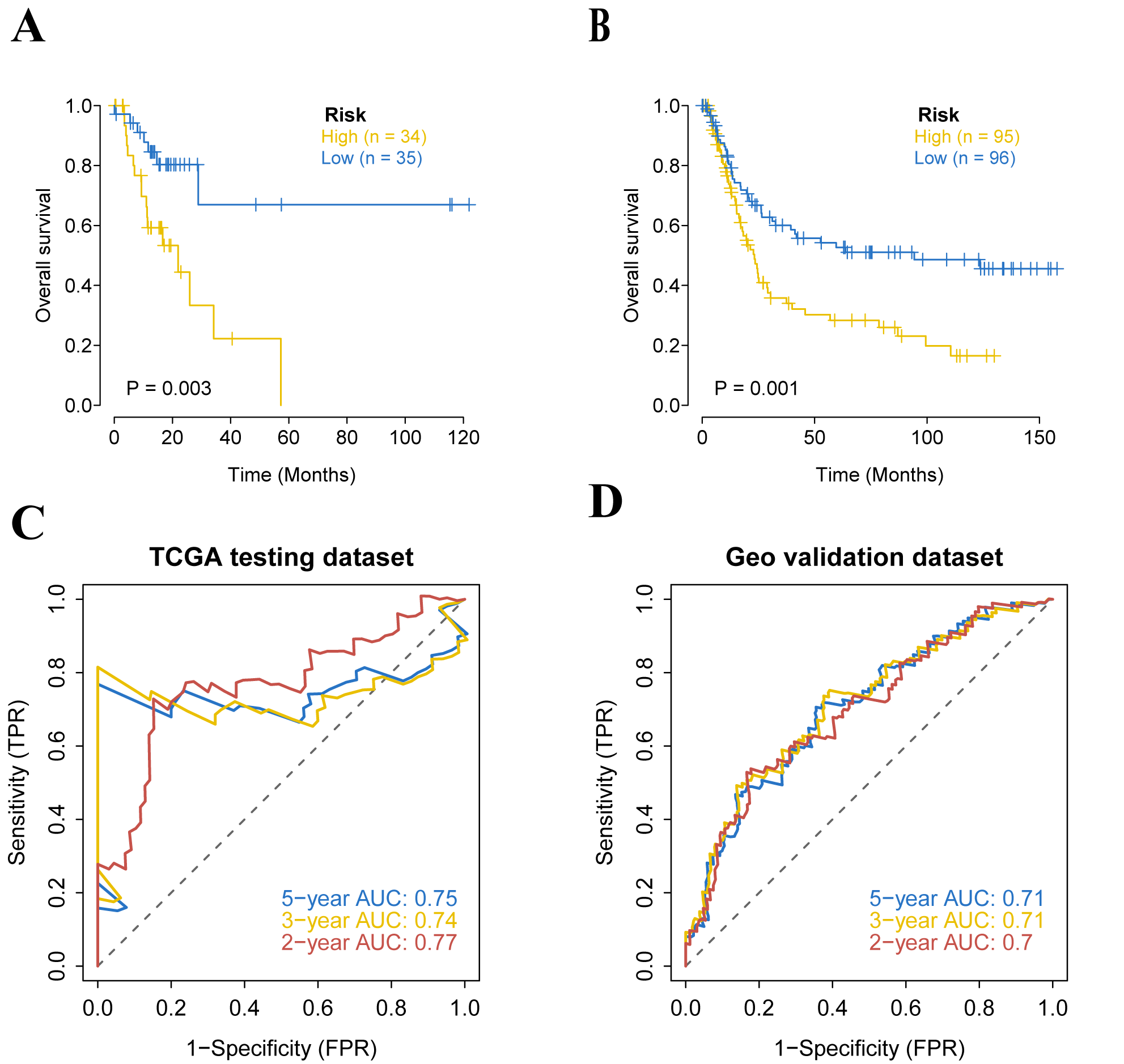

Supplement: Supplementary file 1 [file DataSheet1.ZIP › Fig6.tif]

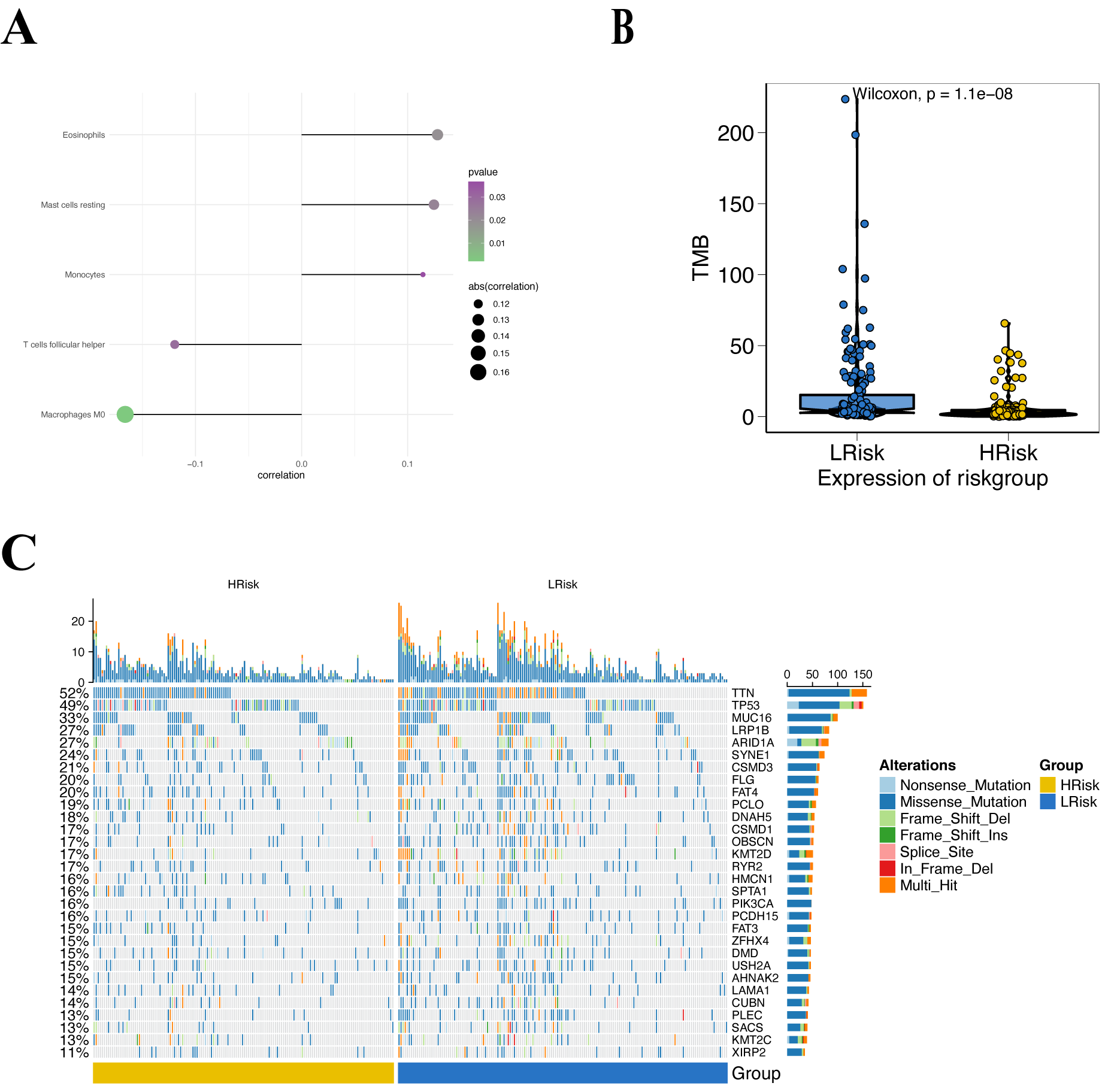

Supplement: Supplementary file 1 [file DataSheet1.ZIP › Fig7.tif]

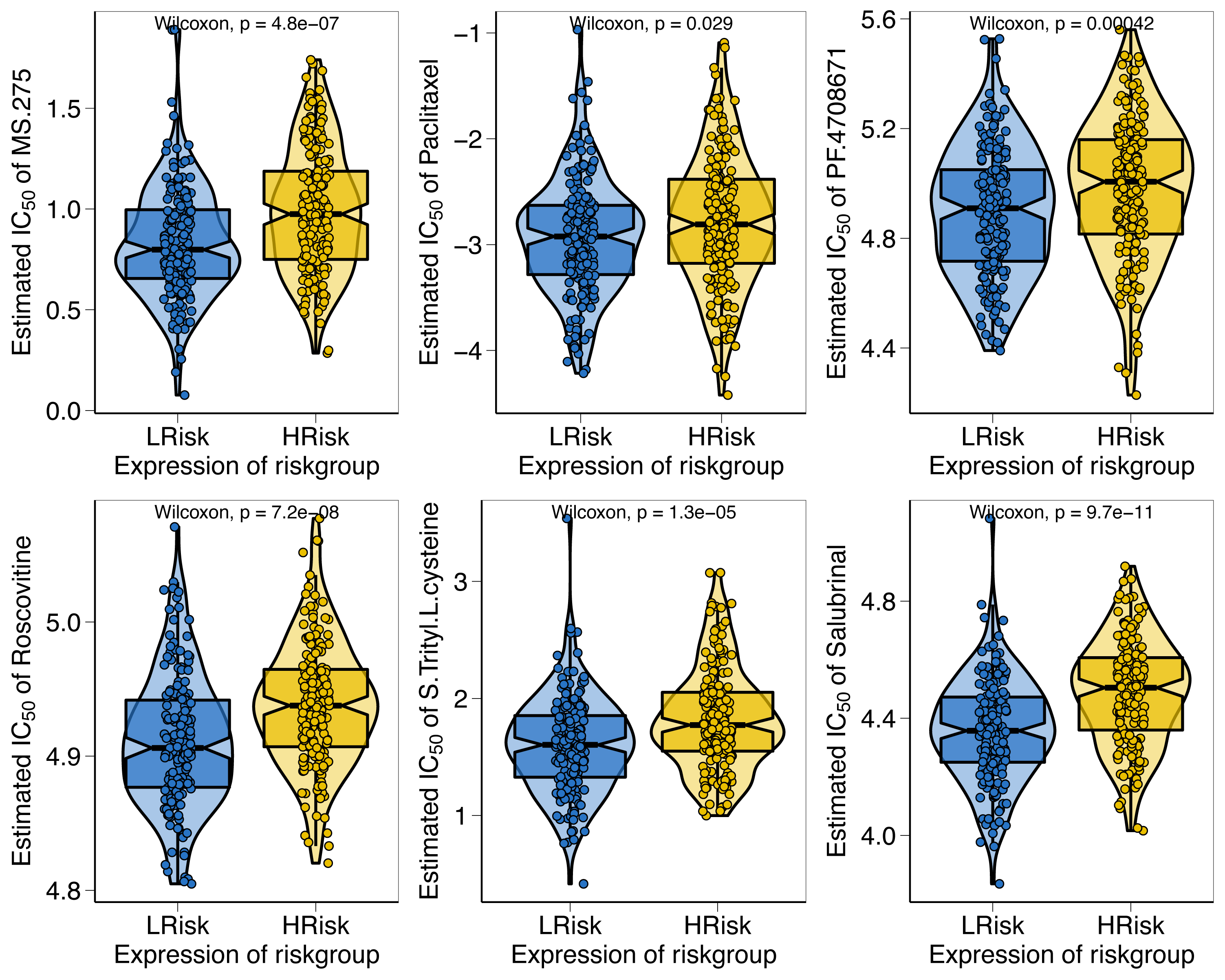

Supplement: Supplementary file 1 [file DataSheet1.ZIP › Fig8.tif]

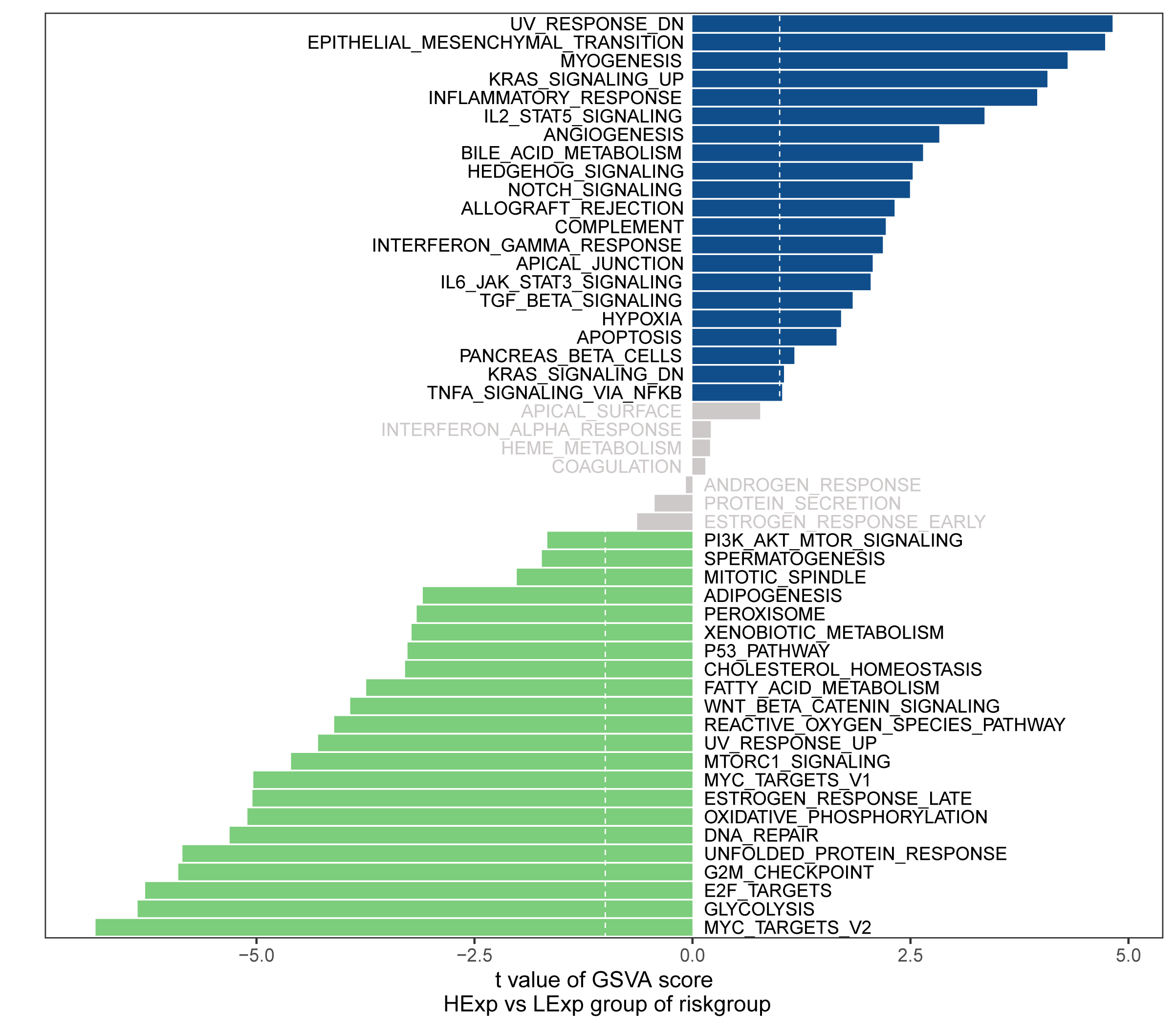

Supplement: Supplementary file 1 [file DataSheet1.ZIP › Fig9.tif]
